# Thioamide substitution to probe the hydroxyproline recognition of VHL ligands

**DOI:** 10.1016/j.bmc.2018.03.034

**Published:** 2018-07-15

**Authors:** Pedro Soares, Xavier Lucas, Alessio Ciulli

**Affiliations:** Division of Biological Chemistry and Drug Discovery, School of Life Sciences, University of Dundee, Dow Street, Dundee DD1 5EH, Scotland, UK

**Keywords:** Protein-ligand interactions, VHL ligands, Thioamides, *n → π^∗^* interaction, PROTACs

## Abstract

Thioamide substitution influences hydrogen bond and *n → π^∗^* interactions involved in the conformational stability of protein secondary structures and oligopeptides. Hydroxyproline is the key recognition element of small molecules targeting the von Hippel-Lindau (VHL) E3 ligase, which are of interest as probes of hypoxia signaling and ligands for PROTAC conjugation. We hypothesized that VHL ligands could be a privileged model system to evaluate the contribution of these interactions to protein:ligand complex formation. Herein we report the synthesis of VHL ligands bearing thioamide substitutions at the central hydroxyproline moiety, and characterize their binding by fluorescence polarization, isothermal titration calorimetry, X-ray crystallography and molecular modeling. In spite of a conserved binding mode, the substitution pattern had a pronounced impact on the ligand affinities. Together the results underscore the role of hydrogen bond and *n → π^∗^* interactions in fine tuning hydroxyproline recognition by VHL.

## Graphical abstract

**Figure:**
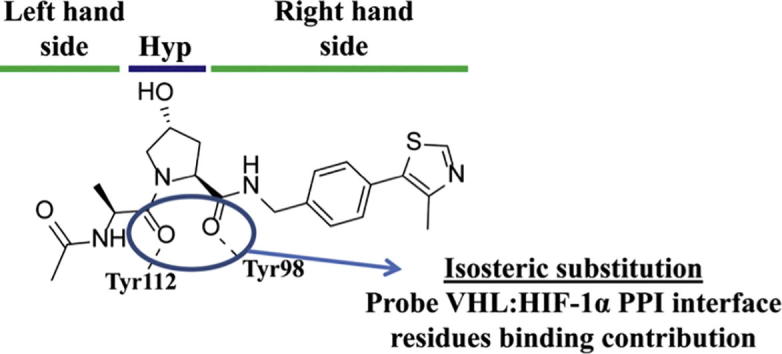

Thioamide is a well-known amide isosteric replacement.^1^ Despite the subtle differences between the two groups (amide oxygen converted into a sulfur in thioamides), they present distinct structural and biological properties.^1^ The length of the C=S bond (∼1.65 Å) is known to be longer than the C=O bond (∼1.19–1.25 Å) due to the larger size of the sulfur atom.^2^, ^3^ Additionally in thioamides the N—H group acts as a stronger hydrogen bond donor than in its amide congener, and *vice versa* the sulfur acts as a weaker hydrogen bond acceptor than the oxygen carbonyl.^4^ In fact, previous studies revealed that the hydrogen bond interaction in O is dominated by a charge-charge attraction, whereas in S the interaction is mainly stabilized by a weaker charge-quadrupole.^5^ The O-to-S substitution has been widely applied to investigate the contribution of hydrogen bond formation to protein secondary structures.^6^, ^7^, ^8^, ^9^, ^10^, ^11^ In contrast, thioamide replacement to probe protein–ligand interactions remains understudied.

In addition to affecting hydrogen bonding, thioamide substitution also modulates the strength of the *n → π^∗^* interaction,^1^, ^12^, ^13^ which has been identified in several systems, including proteins and small molecules.^1^, ^10^, ^12^, ^13^, ^14^, ^15^, ^16^ In this interaction the lone pair (*n*) of a donor group (carbonyl oxygen or thiocarbonyl sulfur) overlaps with the antibonding orbital (*π*^∗^) of an acceptor group (a second carbonyl or thiocarbonyl). This overlap is maximized when the donor and acceptor groups form a short contact in the Bürgi–Dunitz trajectory (∼107°, Fig. 1A).^12^, ^15^, ^17^ Conversion of the donor group from O to a softer base such as S increases the *n → π^∗^* electronic delocalization.^12^ Hence thioamides are better electron-pair donors, increasing the strength of the interaction.^12^

**Fig. 1:**
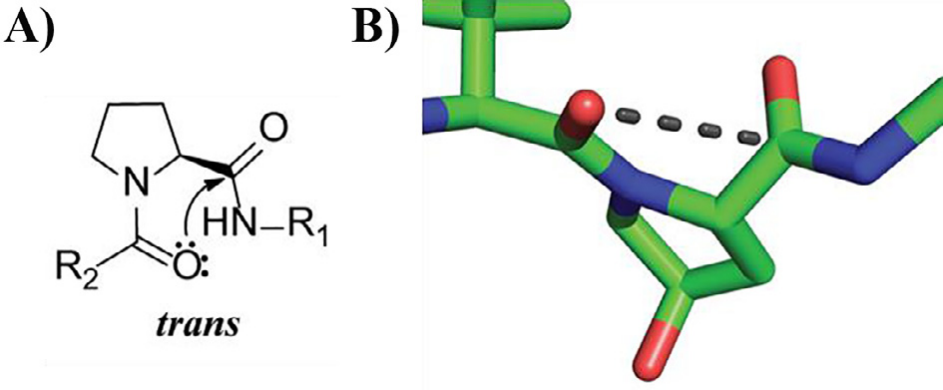
n → π^*^ interaction in prolines and VHL inhibitors. A) Notion of proline backbone carbonyl-carbonyl n → π^*^ interaction. B) Crystal structure of VBC (omitted) in complex with inhibitor VH032 (green carbons) (PDB 4W9H). Possible n → π^*^ interaction in VHL ligands is shown as a black dashed line.

In recent years a series of potent, selective and cell-active inhibitors of the von Hippel-Lindau (VHL) E3 ubiquitin ligase have been designed and optimized around a key hydroxyproline (Hyp) anchor fragment.^18^, ^19^, ^20^, ^21^ VHL inhibitors act on their own as chemical probes of hypoxia signaling.^22^ In addition, VHL ligands are widely used as E3 ligase ligands for PROTAC (proteolysis-targeting chimera) conjugation, a strategy for targeted protein degradation.^23^, ^24^, ^25^, ^26^, ^27^, ^28^ The modified Hyp residue is crucial also for the recognition of the hypoxia inducible factor 1 alpha subunit (HIF-1α), the natural substrate of VHL.^29^, ^30^ A closer analysis of the X-ray co-crystal structure of VHL, elongin B, elongin C (VBC) in complex with inhibitor VH032 (PDB 4W9H)^20^ and HIF-1α peptide (PDB 4AJY)^19^, suggested a conformation compatible with an *n → π^∗^* interaction between the two amide carbonyl groups around the hydroxyproline core (Fig. 1B). We therefore hypothesized that this protein–ligand system could be probed through thioamide substitution.

We began by designing a series of thioamide derivatives of the model VHL ligand **1**,^21^ bearing single as well as double O-to-S substitutions (Fig. 2). The general procedure for the synthesis of thioamide derivatives **2–4** is summarized in Scheme 1 and briefly described in the following paragraph (see Supporting information for detailed synthesis protocols and Supp. Figs. 1–3 for NMR spectra).

**Fig. 2:**
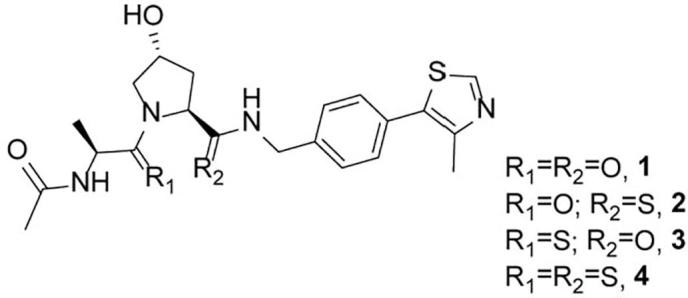
Chemical structure of inhibitor **1** and thioamide derivatives **2–4**.

**Scheme 1:**
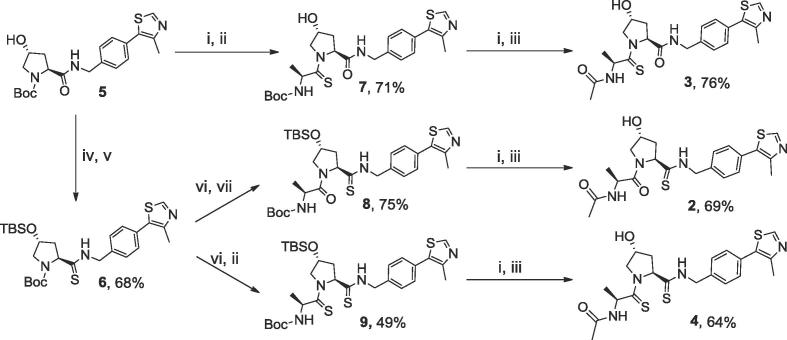
Synthesis of thioamide compounds **2–4**. (i) TFA:DCM (3:7), r.t., 1 h; (ii) activated thioamide (**A**), DIPEA, DMF, r.t., 3 h; (iii) acetic anhydride, Et_3_N, DCM, r.t., 3 h; (iv) TBSCl, imidazole, DMF, r.t., o.n.; (v) ammonium *O*,*O*'-diethyl dithiophosphate, toluene, reflux, o.n.; (vi) TFA:DCM (1:9), r.t., 2 h; (vii) Boc-Ala-OH, HATU, DIPEA, DMF, r.t., 2 h.

The thioamide derivatives **2–4** were obtained from intermediate **5**, which was synthesized as previously described.^20^ Boc deprotection of **5** followed by amide coupling with thioamide derivative **A** ((*S*)-*tert*-Butyl (1-(6-nitro-1*H*-benzo[*d*][1,2,3]triazol-1-yl)-1-thioxopropan-2-yl)carbamate, prepared according to literature^31^) led to intermediate **7** (71%). Boc deprotection of this intermediate followed by acylation yielded the final compound **3** (76%), bearing a single thioamide at the left-hand side. To introduce the O-to-S substitution at the right-hand side, the Hyp hydroxyl group of intermediate **5** was first protected with TBSCl, followed by thioamide conversion to give intermediate **6** (68%). Subsequent Boc deprotection of **6** and HATU (1-[Bis(dimethylamino)methylene]-1*H*-1,2,3-triazolo[4,5-*b*]pyridinium 3-oxid hexafluorophosphate)-assisted amide coupling with Boc-protected alanine led to **8** (75%). Boc deprotection of **8** followed by acylation yielded the final thioamide **2** (69%). To obtain the final doubly substituted compound, deprotected intermediate **6** was reacted with thioamide derivative **A** to give intermediate **9** (49%). Boc deprotection of this intermediate followed by acylation led to the final thioamide derivative **4** (64%).

We also attempted to introduce the thioamide substitution on a more potent analogue of compound **1**, the VHL inhibitor VH032, which bears a *tert-*leucine moiety instead of an alanine.^20^, ^21^, ^22^ However the conversion of the amide on the left-hand side of the hydroxyproline core was not accomplished. In fact, as observed by Engel-Andreasen et al.,^32^ the presence of the hindered *tert*-butyl group near a carbonyl could prevent its conversion to thiocarbonyl.

The binding affinity of the newly synthesized compounds to VHL was evaluated by two orthogonal assays: a direct binding assay using isothermal titration calorimetry (ITC) and a competition assay using fluorescence polarization (FP), which monitored the compound’s ability to displace a fluorescently-labeled high-affinity HIF peptide (Table 1). The results from both assays revealed an excellent agreement on the compounds affinity rank (Fig. 3A and B). The highest affinity compound **3**, which bears a single thioamide conversion at the left-hand side, revealed comparable binding to the unmodified ligand **1**, with only a small twofold loss in binding affinity, as measured by both techniques. In contrast, ligand **2** (single right-hand side thioamide) showed a greater loss in binding affinity (10-fold loss by FP, and 20-fold loss by ITC). Double substitution in ligand **4** resulted in the weakest binder of this series, with *K*_d_ > 10 μM.

**Table 1:** SAR results and computational data of compounds **1–4**. FP back calculated *K*_d_s, ITC measured *K*_d_s and Δ*H*; % of observed *trans* and *cis* isomers in solution measured by NMR; stabilization energy of the n → π^*^ interaction quantified by DFT calculations in model compounds **10–13** (see Supp. Fig. 6); and estimation of interaction energies of the VHL:compound complexes and destabilization of pocket residues Tyr98 and Tyr112 upon binding the thioamide derivatives, as quantified by MM-GBSA calculations.

Compound	*K*_d_ FP (μM)	*K*_d_ ITC (μM)	Δ*H* (kcal/mol)	*trans*:*cis* (%)	E_n→π*_ (kcal/mol)	*E*_MM-GBSA_ (kcal/mol)	Rel. *E*_MM-GBSA_ of Tyr98 (kcal/mol)	Rel. *E*_MM-GBSA_ of Tyr112 (kcal/mol)
1	0.69 ± 0.03	0.44 ± 0.04	−9.12 ± 0.07	92:8	2.1	−110.9	–	–
2	7.07 ± 0.10	9.4 ± 0.3	−5.37 ± 0.05	89:11	2.0	−103.7	2.3	0.0
3	1.0 ± 0.4	0.76 ± 0.02	−6.79 ± 0.01 kcal/mol	94:6	2.6	−104.9	0.1	0.1
4	>13	21.6 ± 0.8	−2.56 ± 0.01	90:10	3.4	−98.4	3.3	0.8

**Fig. 3:**
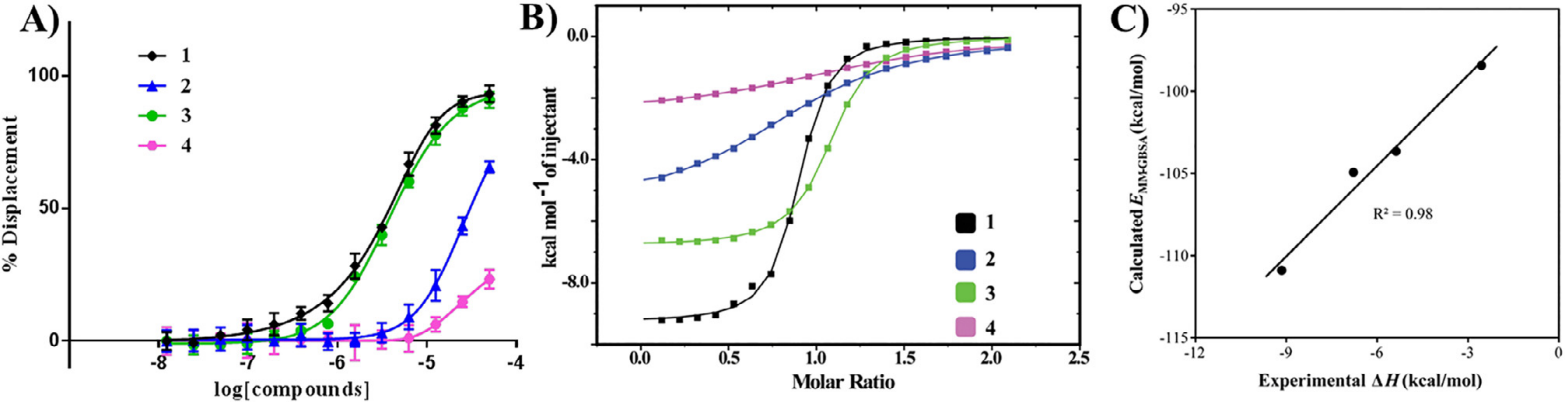
Biophysical characterization of compounds **1–4** binding to VHL. A) Competitive fluorescence polarization binding assay curve of compounds displacing a 20-mer FAM-labeled HIF-1α peptide bound to VBC (*K*_d_ = 3 nM). B) ITC direct titration of compounds into VBC protein complex. C) Predicted *E*_MM-GBSA_ versus experimental Δ*H* for compounds **1–4** binding to VBC.

We next evaluated the impact of thioamide substitutions to the *n → π^∗^* interaction in the ligand free state in solution. This interaction is only possible when the Hyp (thio)amide group is in the *trans* isomer conformation (Fig. 1A). Thus, the ratio between the *trans* and *cis* isomers at equilibrium can be used to infer the strength of the *n → π^∗^* interaction.^13^ Since both *cis* and *trans* isomers of proline backbone amides are populated at room temperature, they can be monitored by NMR spectroscopy due to their slow interconversion, as demonstrated by Newberry et. al.^13^ The NMR results (Table 1) revealed a small decrease on the *trans* isomer when the acceptor group was substituted by a thioamide (**2**) and *vice versa* a small increase of this isomer was observed when the substitution was made on the donor group (**3**). This results support the stronger *n → π^∗^* donor character of thioamides when compared with amides.^12^, ^13^ However, in contrast to what is observed for simple proline models^13^, ^14^, only modest differences in *trans* isomer stabilization in compounds **2** and **3** could be observed (between ±2–3%).

We performed density functional theory (DFT) calculations of the *n → π^∗^* contribution in model compounds **10–13**, in which the substituents at the right- and left-hand side of inhibitors **1–4** were omitted for simplicity (see Supp. Figs. 6 and 7). The results, summarized in Table 1 (see Supp. Table 2 for full details), show that the donor sulfur compound **12** presents an *n → π^∗^* stabilization energy (*E_n→π∗_*) of 2.6 kcal/mol, higher than the parent derivatives **10** and **11** and in good agreement with the NMR results. The *n → π^∗^* contribution in compound **13**, in which both carbonyls are converted to thiocarbonyls, is further increased to 3.4 kcal/mol, indicating a better overlap of the corresponding molecular orbitals in this compound, and consistent with previous calculations by Newberry et al.^13^ Those results would suggest that **4** should have the highest population of *trans* isomer among the studied compounds. Conversely, full thioamide replacement in compound **1** to yield compound **4** led to an unexpected 2% decrease on the *trans* isomer as observed by NMR. Therefore, the conformational preferences of larger compounds **1–4** cannot be reliably studied using surrogate model compounds **10–13**. Importantly, the *trans*:*cis* ratios as observed by NMR did not correlate with binding affinities. This motivated us to carry out a detailed crystallographic analysis of the VHL:inhibitor interactions to provide structural insights on the observed differences in binding affinities.

We obtained high-resolution X-ray crystal structures of compounds **2–4** in complex with VBC (see Supp. Fig. 4, see Supp. Table 1 for data collection and refinement statistics). The omit difference electron density (*F*_o_–*F*_c_) observed unambiguously identified the inhibitor bound at the expected VHL site (Fig. 4A and Supp. Fig. 5). The new structures revealed that the noncovalent interaction network between the compounds and VHL residues was fully conserved and consistent with those observed with previous inhibitors (Fig. 4B).^20^ Superposition of the crystal structures of VBC in complex with compounds **1–4** also revealed that thioamide conversion induced a slight change in the position of the Tyr112 side chain (Fig. 4C). The presence of the thioamide on the left-hand side of Hyp resulted in Tyr112 bending slightly to accommodate the bulkier group. Additionally we measured the hydrogen bond distances between the compounds right-hand side O/S acceptor groups and the side chain hydroxyl of Tyr98. An increase in hydrogen bond distances was observed for compounds **2** and **4** (3.0 ± 0.1 and 2.95 ± 0.05 Å for O–H⋯S=C groups, respectively) compared to **1** and **3** (2.52 ± 0.05 Å for O–H⋯O=C groups, on both compounds). This trend could be explained by the increased van der Walls radius and decreased electronegativity of sulfur compared to oxygen^5^, and is consistent with the thioamide group being a weaker hydrogen bond acceptor compared to the amide group.^33^ Together, our data reveal a key role of the Tyr98 side chain hydrogen bond in VHL ligand binding.

**Fig. 4:**
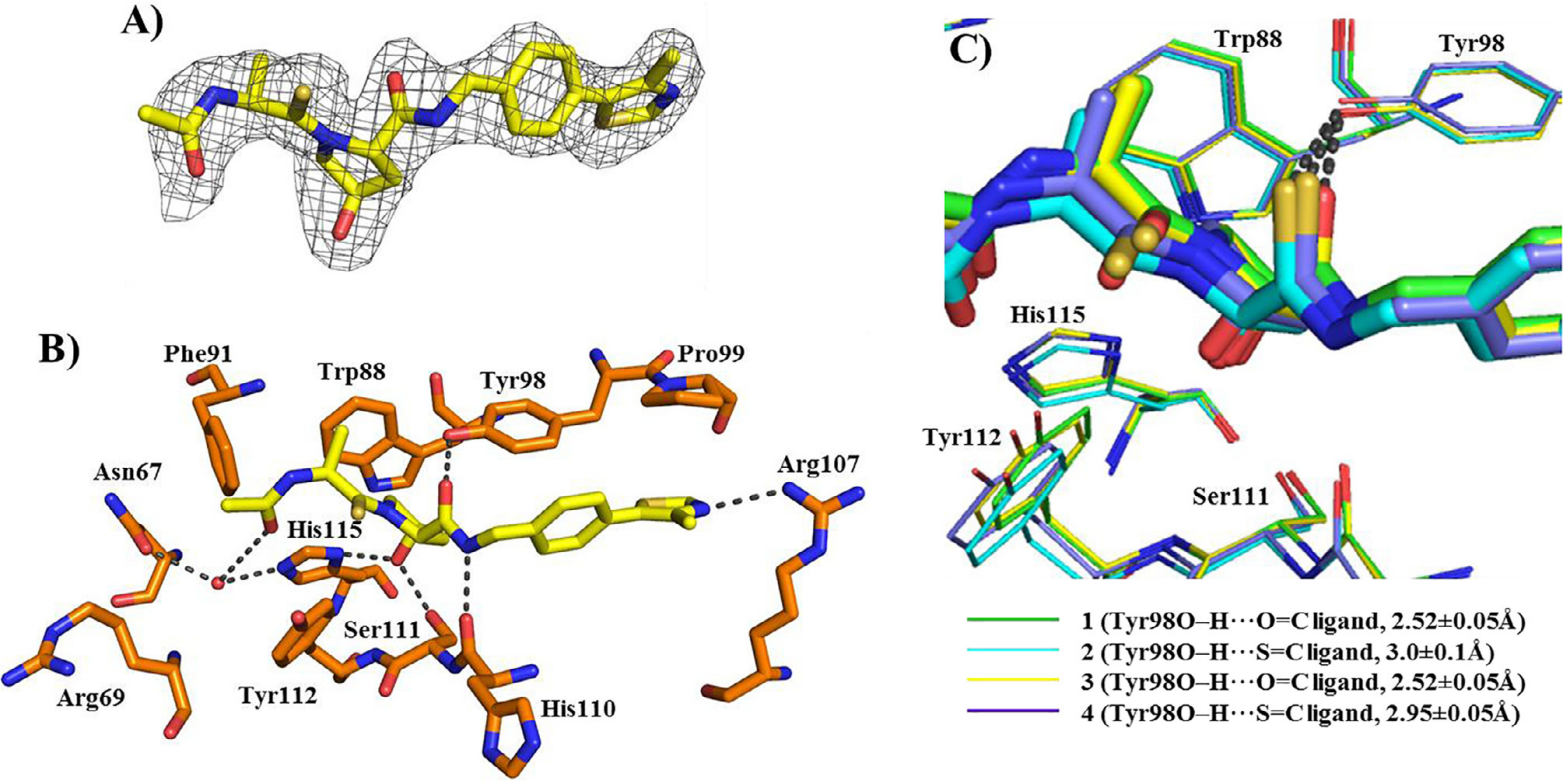
Co-crystal structures of compounds **1–4** in complex with VBC. A) The omit difference electron density (*F_o_-F_c_*) superimposed around **3** is shown in blue contoured at 3σ and with a 2.0 Å carve radius. B) Detailed binding interactions of compound **3** (yellow carbons) with VHL pocket residues. VHL residues forming the binding pocket are shown as orange stick representations. Water forming hydrogen bond with the compound is shown as a red sphere. Hydrogen bond interactions between compound, bound waters and VHL pocket residues are shown as black dashed lines. C) Superposition of VBC structures in complex with compounds **1** (green carbons), **2** (light blue carbons), **3** (yellow carbons) and **4** (purple carbons) showing details of VHL binding pocket. Residues around hydroxyproline core are presented with the same color as the respective bound compounds. Hydrogen bond interaction between compounds and Tyr98 is shown as black dashed lines.

We next performed molecular mechanics calculations on each of the ligand:VHL complexes using the generalized-Born surface area (MM-GBSA) approximation and estimated interaction energies (*E*_MM-GBSA_) between the ligands and the protein. The predicted *E*_MM-GBSA_ values are in excellent agreement with experimental enthalpic contributions to binding (Δ*H*, Fig. 3C), and in good agreement with Gibbs free energies (Δ*G*, Supp. Fig. 8). We therefore evaluated the interaction energy of each protein amino acid in the complexes with its surrounding to determine differences in relative *E*_MM-GBSA_ compared to reference inhibitor **1**. The results, shown in Table 1, indicate that the loss in binding affinity observed in compounds **2** and **4** is largely contributed by destabilization of Tyr98 and, to a much lesser extent, of Tyr112, especially in compound **4**. Thus, the molecular modeling calculations provided a solid computational model to predict the impact of O-to-S substitutions of amides in binding affinity and enabled quantification of the subtle structural changes observed in the crystal structures of VHL in complex with the inhibitors.

In summary we describe the synthesis and biophysical characterization of a series of thioamide derivatives of a potent VHL inhibitor. In spite of a fully conserved binding mode, the pattern of substitution had markedly varying effects on binding affinities. Substitution at the left-hand side amide was tolerated, while substitution at the right-hand side had the most detrimental effect, highlighting the prevalent role of the Tyr98 hydrogen bond in molecular recognition. Molecular modeling calculations could recapitulate the trends in binding affinities observed experimentally and provided a theoretical framework for understanding the subtle structural changes observed crystallographically. The results of this study could prove useful to future drug design of VHL inhibitors for PROTACs. More generally, we provide a combined biophysical, structural and modeling characterization cascade that could be applied to study the role of thioamide substitutions in other protein–ligand interaction systems.

## Accession codes

PDB accession codes of VBC in complex with **2–4** are 6FMI, 6FMJ and 6FMK, respectively. Authors will release the atomic coordinates upon article publication.
