# Supplementary material for: Thioamide substitution to probe the hydroxyproline recognition of VHL ligands
Source: Bioorg Med Chem. 2018 Jul 15;26(11):2992–5. doi: 10.1016/j.bmc.2018.03.034 (PMC6008493; doi:10.1016/j.bmc.2018.03.034)
Supplement: Supplementary data 1 [file mmc1.docx]

**Thioamide substitution to probe the hydroxyproline recognition of VHL ligands**

**Pedro Soares, Xavier Lucas, Alessio Ciulli^*^**

Division of Biological Chemistry and Drug Discovery, School of Life Sciences, University of Dundee, Dow Street, Dundee, DD1 5EH, Scotland, UK.

**Supporting Information**

**Table of Contents**

**S2 – Thioamide inhibitor synthesis and characterization**

**S13 – FP competition assay protocol and data**

**S15 – ITC protocols and data**

**S18 - Quantification of ligands *trans* and *cis* isomers in solution**

**S18 – X-ray crystallography protocol, data processing and refinement statistics**

**S21 – Computational methods**

1. **Thioamide inhibitor synthesis and characterization**

**General.** Commercially available starting reagents for each reaction were purchased from Sigma Aldrich, Fluorochem, Apollo Scientific or Alfa Aesar and used without further purification. All reactions were carried using anhydrous solvents. Analytical thin-layer chromatography (TLC) was performed on pre-coated TLC plates (layer 0.20 mm silica gel 60 with fluorescent indicator (UV 254: Merck)). The TLC plates were air dried and revealed under UV lamp (254/365 nm). Flash-column chromatography was performed in pre-packed silica gel cartridges (230-400 mesh, 40-63 mm; SiliCycle) using a Teledyne ISCO Combiflash Companion or Combiflash Retrieve using the solvent mixtures stated for each synthesis as mobile phase.

Liquid chromatography-mass spectrometry (LC-MS) analyses were performed with either an Agilent HPLC 1100 series connected to a Bruker Daltonics MicroTOF or an Agilent Technologies 1200 series HPLC connected to an Agilent Technologies 6130 quadrupole spectrometer or a Waters 2795 connected to a Waters ZQ Micromass spectrometer, where all instruments were connected to a diode array detector. All the final compounds used in all the experiments were evaluated after preparative LC-MS separations with a Waters X-bridge C18 column (50 mm x 2.1 mm x 3.5 mm particle size); flow rate, 0.5 mL/min with a mobile phase of water/MeCN+0.1% CHOOH or water/MeCN+0.1% NH_3_; 95/5 water/MeCN was initially held for 0.5 min followed by a linear gradient from 95/5 to 5/95 water/MeCN over 3.5 min which was then held for 2 min. The purity of all the compounds was evaluated using the analytical LC-MS system as described above, and yielded a purity >95%.

High-resolution electrospray measurements were performed on a Bruker Daltonics MicroTOF mass spectrometer. ^1^H NMR and ^13^C NMR spectra were recorded on a Bruker DPX-400 Cryo spectrometer (^1^H at 400.1 MHz; ^13^C at 101 MHz). Chemical shifts (δ) are expressed in ppm reported using residual solvent as the internal reference in all cases. Signal splitting patterns are described as singlet (s), doublet (d), triplet (t), quartet (q), multiplet (m), or a combination thereof. Coupling constants (*J*) are quoted to the nearest 0.1 Hz.

Intermediate **5** and inhibitor **1** were synthesized as described elsewhere.^1–3^ Activated thioamide derivative **A** was prepared according to the literature protocol.^4^

**(*S*)-*tert*-butyl (1-((2-amino-5-nitrophenyl)amino)-1-oxopropan-2-yl)carbamate**

To a solution of 4-nitrobenzene-1,2-diamine (500 mg, 3.26 mmol) in DMF was added Boc-Ala-OH (617 mg, 3.26 mmol, 1 equiv.). DIPEA (1.26 g, 1.67 mL, 9.78 mmol, 3 equiv.) was added dropwise, and the mixture was stirred for 5 min at room temperature. HATU (2.48 g, 6.52 mmol, 2 equiv.) was then added and the reaction left to stir at room temperature for 3h. Water was added, and the mixture was extracted with ethyl acetate (3x). The combined organic phases were washed with brine, dried over anhydrous MgSO_4_ and evaporated to afford the corresponding crude compound that was purified by flash column chromatography using a gradient elution of 10% to 80% ethyl acetate in heptane to yield the final compound as a pale yellow solid (846 mg, 2.60 mmol, 80%), which spectral data matched the one published before.^5^ ^1^H NMR (DMSO-*d_6_*, 400 MHz): δ 9.37 (s, 1H), 8.10 (d, 1H, *J* = 2.1 Hz), 7.87 (dd, 1H, *J* = 9.0, 2.6 Hz), 7.21 (d, 1H, *J* = 6.2 Hz), 6.76 (d, 1H, *J* = 9.0 Hz), 6.46 (s, 2H), 4.14-4.07 (m, 1H), 1.39 (s, 9H), 1.28 (d, 3H, *J* = 7.1 Hz). HRMS (ESI) m/z: [M^+^+1] calculated for C_14_H_20_N_4_O_5_S_2_: 324.1434; observed: 324.1430.

**(*S*)-*tert*-butyl (1-((2-amino-5-nitrophenyl)amino)-1-thioxopropan-2-yl)carbamate**

To a solution of (*S*)-*tert*-butyl (1-((2-amino-5-nitrophenyl)amino)-1-oxopropan-2-yl)carbamate (700 mg, 2.16 mmol) in dry THF was added the Lawesson’s reagent (660 mg, 1.62 mmol, 0.75 equiv.) and the mixture was refluxed for 2 h. An additional portion of Lawesson’s reagent (660 mg, 1.62 mmol, 0.75 equiv.) was then added and mixture was refluxed overnight. The solvent was evaporated and the crude compound was purified by column chromatography using a gradient elution of 10% to 100% ethyl acetate in heptane to yield the final compound as a yellow solid (367 mg, 1.07 mmol, 50%), which spectral data matched the one published before.^5^ ^1^H NMR (DMSO-*d_6_*, 400 MHz): δ 10.20 (s, 1H), 7.96 (dd, 1H, *J* = 9.1, 2.6 Hz), 7.84 (s, 1H), 7.43 (d, 1H, *J* = 5.0 Hz), 6.77 (d, 1H, *J* = 9.0 Hz), 6.46 (s, 2H), 4.46-4.40 (m, 1H), 1.39 (s, 9H), 1.38 (d, 3H, *J* = 6.6 Hz). HRMS (ESI) m/z: [M^+^+1] calculated for C_14_H_20_N_4_O_4_S: 340.1205; observed: 340.1207.

**(*S*)-*tert*-Butyl (1-(6-nitro-1*H*-benzo[*d*][1,2,3]triazol-1-**yl**)-1-thioxopropan-2-yl)carbamate (A)**

To a solution of (*S*)-*tert*-butyl (1-((2-amino-5-nitrophenyl)amino)-1-thioxopropan-2-yl)carbamate (0.300 mg, 0.88 mmol) in a mixture of acetic acid:water (95:5) at 4ºC was added sodium nitrite (97 mg, 1.41 mmol, 1.5 equiv.). After stirring the mixture for 30 min at 4ºC, chilled water was added and the precipitate then formed was filtered and dried to yield the final compound as a pale yellow solid (0.279 mg, 0.79 mmol, 90%), which spectral data matched the one published before.^5^ ^1^H NMR (CDCl_3_): δ 9.61 (s, 1H), 8.46 (d, 1H, *J* = 9.1 Hz), 6.23-6.17 (m, 1H), 5.43 (d, 1H, *J* = 8.5 Hz), 1.64 (d, 3H, 6.9 Hz), 1.40 (s, 9H). HRMS (ESI) m/z: [M^+^+1] calculated for C_14_H_17_N_5_O_4_S: 351.1001; observed: 351.1003.

**(2*S*,4*R*)-*tert*-Butyl 4-((*tert*-butyldimethylsilyl)oxy)-2-((4-(4-methylthiazol-5-yl)benzyl)**

**carbamothioyl)pyrrolidine-1-carboxylate (6)**

To a solution of **5** (500 mg, 1.20 mmol) in dry DMF was added TBSCl (360 mg, 2.40 mmol, 2 equiv.) and imidazole (326 mg, 4.79 mmol, 4 equiv.) and the reaction mixture was then stirred overnight under N_2_. Water was added, and the mixture was extracted with ethyl acetate (3x). The combined organic phases were washed with brine, dried over anhydrous MgSO_4_ and evaporated to afford the corresponding TBS protected compound as a colorless oil which was used in the following reaction without further purification.

To a solution of the TBS protected compound (637 mg, 1.20 mmol) in toluene was added ammonium *O*,*O*'-diethyl dithiophosphate (473 mg, 2.39 mmol, 2 equiv.) and the reaction mixture was then refluxed overnight. Solvent was removed under reduced pressure and the obtained residue was partitioned between water and ethyl acetate and after collection of the organic layer the aqueous phase was extracted again with ethyl acetate (2x). The combined organic phases were washed with brine, dried over anhydrous MgSO_4_ and evaporated to afford the corresponding crude compound that was purified by flash column chromatography using gradient elution of 0% to 80% of ethyl acetate in heptane to yield the final compound as a white solid (447 mg, 0.82 mmol, 68%). mp 42–46 °C; ^1^H NMR (CDCl_3_, 400 MHz): δ 8.69 (s, 1H), 7.43-7.37 (m, 4H), 5.13-4.69 (m, 3H), 4.50-4.32 (m, 1H), 3.76-3.46 (m, 2H), 3.30 (d, 2H, *J* = 7.9 Hz), 2.53 (s, 3H), 3.32-3.28 (m, 1H), 2.26-2.05 (m, 1H), 1.40 (s, 9H), 0.87 (s, 9H), 0.08 (s, 3H), 0.09 (s, 3H); ^13^C NMR (CDCl_3_, 101 MHz): δ 203.9, 188.6, 150.7, 149.0, 136.3, 129.9, 128.7, 81.4, 70.6, 65.4, 55.8, 49.5, 44.3, 41.1, 28.5, 26.0, 18.2, 16.4, -4.6. HRMS (ESI) m/z: [M^+^+1] calculated for C_27_H_42_N_3_O_3_S_2_Si: 548.2431; observed: 548.2450.

***tert*-Butyl ((*S*)-1-((2*S*,4*R*)-4-hydroxy-2-((4-(4-methylthiazol-5-yl)benzyl)carbamoyl)**

**pyrrolidin-1-yl)-1-thioxopropan-2-yl)carbamate (7)**

A solution of **5** (130 mg, 0.31 mmol) in TFA:DCM (3:7) was stirred at room temperature for 1h. The solvents were evaporated under reduced pressure to give the corresponding deprotected intermediate (TFA salt) as brown oil which was used in the following reaction without further purification.

The deprotected intermediate (134 mg, 0.31 mmol) was solubilized in DMF and to the resulting solution was added DIPEA (161 mg, 210 µL, 1.24 mmol, 4 equiv.) and the resulting mixture was stirred for 10 min at room temperature. Activated thioamide derivative **A** (164 mg, 0.47 mmol, 1.5 equiv.) was added and the resulting mixture was further stirred for 3h at room temperature. Water was added, and the mixture was extracted with ethyl acetate (3x). The combined organic phases were washed with brine, dried over anhydrous MgSO_4_ and evaporated and evaporated to afford the corresponding crude compound that was purified by flash column chromatography using a gradient of 10% to 70% acetone in heptane to yield to yield compound **7** as a white solid (108 mg, 0.21 mmol, 71%). mp 67–71 °C; ^1^H NMR (CD_3_OD, 400 MHz): δ 8.88 (s, 1H), 7.48-7.42 (m, 4H), 4.98-4.88 (m, 1H), 4.38 **(**q, 1H, *J* = 6.9 Hz**)**, 3.85 (dd, 1H, *J* = 10.5, 4.1 Hz), 3.75 (d, 1H, *J* = 11.2 Hz), 2.50 (s, 3H), 2.32-2.26 (m, 1H), 2.20-2.14 (m, 1H), 1.41 (s, 9H), 1.31 (d, 3H, *J* = 6.8 Hz); ^13^C NMR (CD_3_OD, 101 MHz): δ 204.8, 173.2, 156.2, 151.6, 147.8, 138.1, 132.2, 130.4, 128.9, 128.1, 78.7, 69.0, 66.0, 55.8, 44.2, 27.1, 25.3, 15.9, 14.3. HRMS (ESI) m/z: [M^+^+1] calculated for C_24_H_32_N_4_O_4_S_2_: 505.1865; observed: 505.1859.

***tert*-Butyl ((*S*)-1-((2*S*,4*R*)-4-((*tert*-butyldimethylsilyl)oxy)-2-((4-(4-methylthiazol-5-yl)**

**benzyl)carbamothioyl)pyrrolidin-1-yl)-1-oxopropan-2-yl)carbamate (8)**

A solution of **6** (200 mg, 0.36 mmol) in TFA:DCM (1:9) was stirred at room temperature for 2 h. The solvents were evaporated under reduced pressure to give the corresponding deprotected intermediate (TFA salt) as a yellowish oil that was used in the following reaction without further purification.

To a solution of the deprotected intermediate (TFA salt, 205 mg, 0.36 mmol) in DMF was added Boc-Ala-OH (69 mg, 0.36 mmol, 1 equiv.). DIPEA (189 mg, 250 µL, 1.46 mmol, 4 equiv.) was added dropwise, and the mixture was stirred for 5 min at room temperature. HATU (149 mg, 0.39 mmol, 1.1 equiv.) was added and the mixture was stirred at room temperature for 2h. Water was added, and the mixture was extracted with ethyl acetate (3x). The combined organic phases were washed with brine, dried over anhydrous MgSO_4_ and evaporated to afford the corresponding crude compound that was purified by flash column chromatography using a gradient of 10% to 70% acetone in heptane to yield compound **8** white solid (169 mg, 0.27 mmol, 75%). mp 57–60 °C; ^1^H NMR (CD_3_OD, 400 MHz): δ 8.87 (s, 1H), 7.47-7.41 (m, 4H), 5.00-4.89 (m, 4H), 4.73-4.68 (m, 1H), 4.37 **(**q, 1H, *J* = 7.0 Hz**)**, 3.84 (dd, 1H, *J* = 10.4, 4.0 Hz**)**, 3.75 (d, 1H, *J* = 11.1 Hz), 2.50 (s, 3H), 2.31-2.25 (m, 1H), 2.19-2.13 (m, 1H), 1.42 (s, 9H), 1.30 (d, 3H, *J* = 7.1 Hz), 0.91 (s, 9H), 0.12 (s, 6H); ^13^C NMR (CD_3_OD, 101 MHz): δ 204.5, 172.9, 156.2, 151.5, 147.7, 137.2, 132.0, 130.4, 129.0, 128.0, 79.1, 69.1, 65.9, 55.8, 44.0, 41.2, 27.3, 24.9, 15.8, 14.4, -6.1. HRMS (ESI) m/z: [M^+^+1] calculated for C_24_H_32_N_4_O_4_S_2_: 505.1865; observed: 505.1859.

***tert*-Butyl ((*S*)-1-((2*S*,4*R*)-4-((*tert*-butyldimethylsilyl)oxy)-2-((4-(4-methylthiazol-5-yl)**

**benzyl)carbamothioyl)pyrrolidin-1-yl)-1-thioxopropan-2-yl)carbamate (9)**

A solution of **6** (200 mg, 0.36 mmol) in TFA:DCM (1:9) was stirred at room temperature for 2 h. The solvents were evaporated under reduced pressure to give the corresponding deprotected intermediate (TFA salt) as a yellowish oil that was used in the following reaction without further purification.

The deprotected intermediate (205 mg, 0.36 mmol) was solubilized in DMF and to the resulting solution was added DIPEA (189 mg, 250 µL, 1.46 mmol, 4 equiv.) and the resulting mixture was stirred for 10 min at room temperature. Activated thioamide derivative **A** (187 mg, 0.53 mmol, 1.5 equiv.) was added and the resulting mixture was further stirred for 3h at room temperature. The combined organic phases were washed with brine, dried over anhydrous MgSO_4_ and evaporated to afford the corresponding crude compound that was purified by flash column chromatography using a gradient of 10% to 70% acetone in heptane to afford compound **9** as a pale yellow solid (113 mg, 0.18 mmol, 49%). mp 64–68 °C; ^1^H NMR (CD_3_OD, 400 MHz): δ 8.89 (s, 1H), 7.51 **(**d, 2H, *J* = 8.5 Hz**)**, 7.48 **(**d, 2H, *J* = 8.3 Hz**)**, 5.01-4.91 (m, 4H), 4.73-4.69 (m, 1H), 4.37 (q, 1H, *J* = 6.8 Hz), 3.84 (dd, 1H, *J* = 11.8, 2.9 Hz), 3.75 (d, 1H, *J* = 11.2 Hz), 2.50 (s, 3H), 2.31-2.25 (m, 1H), 2.19-2.13 (m, 1H), 1.42 (s, 9H), 1.31 **(**d, 3H, *J* = 6.8 Hz), 0.91 (s, 9H), 0.12 (s, 6H); ^13^C NMR (CD_3_OD, 101 MHz): δ 204.5, 203.9, 155.4, 151.3, 148.0, 137.0, 132.0, 130.4, 129.0, 128.0, 79.0, 69.3, 66.0, 56.0, 44.3, 41.0, 27.6, 25.0, 16.0, 14.7, -7.0. HRMS (ESI) m/z: [M^+^+1] calculated for C_30_H_46_N_4_O_3_S_3_Si: 634.2501; observed: 634.2496.

**General acylation protocol.** A solution of Boc protected intermediate (0.19 mmol) in TFA:DCM (3:7) was stirred at room temperature for 1 h. The solvents were evaporated under reduced pressure to give the corresponding deprotected intermediate (TFA salt) as a brown oil that was used in the following reactions without further purification. To a solution of the deprotected intermediate (TFA salt, 0.19 mmol) in DCM was added triethylamine (0.57 mmol, 3 equiv.). After stirring the mixture for 10 min at room temperature, acetic anhydride (0.29 mmol, 1.5 equiv.) was added and the resulting mixture was then stirred 3 h at room temperature. The solvents were evaporated under reduced pressure to afford the corresponding crude compound that was purified by flash column chromatography using a gradient of 10% to 80% acetone in heptane to yield the final compounds as solids.

***N*-((*S*)-1-((2*S*,4*R*)-4-Hydroxy-2-((4-(4-methylthiazol-5-yl)benzyl)carbamothioyl)**

**pyrrolidin-1-yl)-1-oxopropan-2-yl)acetamide (2)**

Following the general acylation protocol, from intermediate **8** (117 mg, 0.19 mmol), triethylamine (57 mg, 79 µL, 0.57 mmol, 3 equiv.) and acetic anhydride (29 mg, 27 μL, 0.29 mmol, 1.5 equiv.), compound **2** was obtained as a white powder (58 mg, 0.13 mmol, 69%). mp 52–54 °C; ^1^H NMR (CD_3_OD, 400 MHz): δ 8.88 (s, 1H), 7.48-7.42 (m, 4H), 5.06 (t, 1H, *J* = 7.7 Hz), 4.96 (q, 1H, *J* = 6.8 Hz), 4.60-4.56 (m, 1H), 4.51 (d, 1H, *J* = 15.4 Hz), 4.37 (d, 1H, *J* = 15.4 Hz), 4.07 (dd, 1H, *J* = 11.8, 2.6 Hz), 3.93 (dd, 1H, *J* = 11.6, 4.8 Hz), 2.48 (s, 3H), 2.35-2.29 (m, 1H), 2.21-2.15 (m, 1H), 1.97 (s, 3H), 1.37 (d, 3H, *J* = 6.7 Hz); ^13^C NMR (CD_3_OD, 101 MHz): δ 205.1, 173.2, 172.4, 152.8, 149.1, 140.2, 133.4, 131.6, 130.3, 129.1, 70.5, 66.5, 59.8, 53.2, 43.7, 38.9, 22.6, 20.7, 15.8. HRMS (ESI) m/z: [M^+^+1] calculated for C_21_H_27_N_4_O_3_S_2_: 447.1519; observed: 447.1527.

**(2*S*,4*R*)-1-((*S*)-2-Acetamidopropanethioyl)-4-hydroxy-*N*-(4-(4-methylthiazol-5-yl)**

**benzyl)pyrrolidine-2-carboxamide (3)**

Following the general acylation protocol, from intermediate **7** (96 mg, 0.19 mmol), triethylamine (57 mg, 79 µL, 0.57 mmol, 3 equiv.) and acetic anhydride (29 mg, 27 μL, 0.29 mmol, 1.5 equiv.), compound **2** was obtained as a white powder (64 mg, 0.14 mmol, 76%). mp 54–56 °C; ^1^H NMR (CD_3_OD, 400 MHz): δ 8.89 (s, 1H), 7.49-7.43 (m, 4H), 5.08 **(**t, 1H, *J* = 7.8 Hz), 4.98 (q, 1H, *J* = 6.8 Hz ), 4.62-4.58 (m, 1H), 4.53 (d, 1H, *J* = 15.4 Hz), 4.39 (d, 1H, *J* = 15.4 Hz), 4.09 (dd, 1H, *J* = 11.8, 2.3 Hz), 3.95 (dd, 1H, *J* = 11.9, 4.7 Hz), 2.50 (s, 3H), 2.37-2.30 (m, 1H), 2.23-2.16 (m, 1H), 1.98 (s, 3H), 1.39 (d, 3H, *J* = 6.9 Hz); ^13^C NMR (CD_3_OD, 101 MHz): δ 205.1, 173.2, 172.4, 152.8, 149.1, 140.2, 133.4, 131.6, 130.3, 129.1, 70.5, 66.5, 59.8, 53.2, 43.7, 38.9, 22.6, 20.7, 15.8. HRMS (ESI) m/z: [M^+^+1] calculated for C_21_H_27_N_4_O_3_S_2_: 447.1519; observed: 447.1520.

***N*-((*S*)-1-((2*S*,4*R*)-4-hydroxy-2-((4-(4-methylthiazol-5-yl)benzyl)carbamothioyl)**

**pyrrolidin-1-yl)-1-thioxopropan-2-yl)acetamide (4)**

Following the general acylation protocol, from intermediate **9** (120 mg, 0.19 mmol), triethylamine (57 mg, 79 µL, 0.57 mmol, 3 equiv.) and acetic anhydride (29 mg, 27 μL, 0.29 mmol, 1.5 equiv.), compound **2** was obtained as a white powder (56 mg, 0.12 mmol, 64%). mp 61–63 °C; ^1^H NMR (CD_3_OD, 400 MHz): δ 8.88 (s, 1H), 7.50 (d, 2H, *J* = 8.6 Hz ), 7.44 (d, 2H, *J* = 8.3 Hz), 5.46 (t, 1H, *J* = 7.5 Hz), 5.04 (d, 1H, *J* = 15.1 Hz) 4.97 (q, 1H, *J* = 6.7 Hz ), 4.77 (d, 1H, *J* = 15.2 Hz), 4.71-4.68 (m, 1H), 4.14 (dd, 1H, *J* = 11.8, 2.2 Hz), 4.04 (dd, 1H, *J* = 11.8, 4.6 Hz), 2.49 (s, 3H), 2.36-2.26 (m, 2H), 1.97 (s, 3H), 1.36 (d, 3H, *J* = 6.8 Hz); ^13^C NMR (CD_3_OD, 101 MHz): δ 205.0, 204.3, 172.3, 152.9, 149.1, 138.6, 133.4, 131.8, 130.3, 129.6, 72.2, 70.7, 60.4, 53.3, 41.6, 22.6, 20.2, 15.8. HRMS (ESI) m/z: [M^+^+1] calculated for C_21_H_27_N_4_O_3_S_3_: 463.1291; observed: 463.1285.


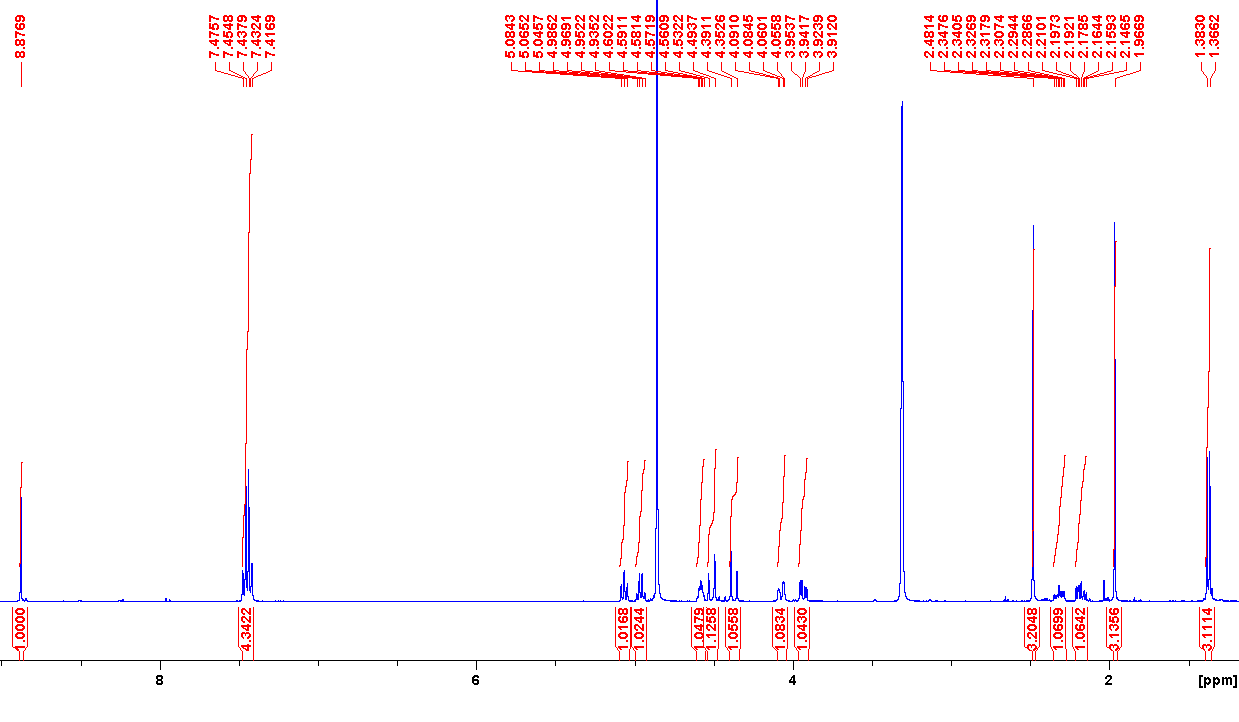


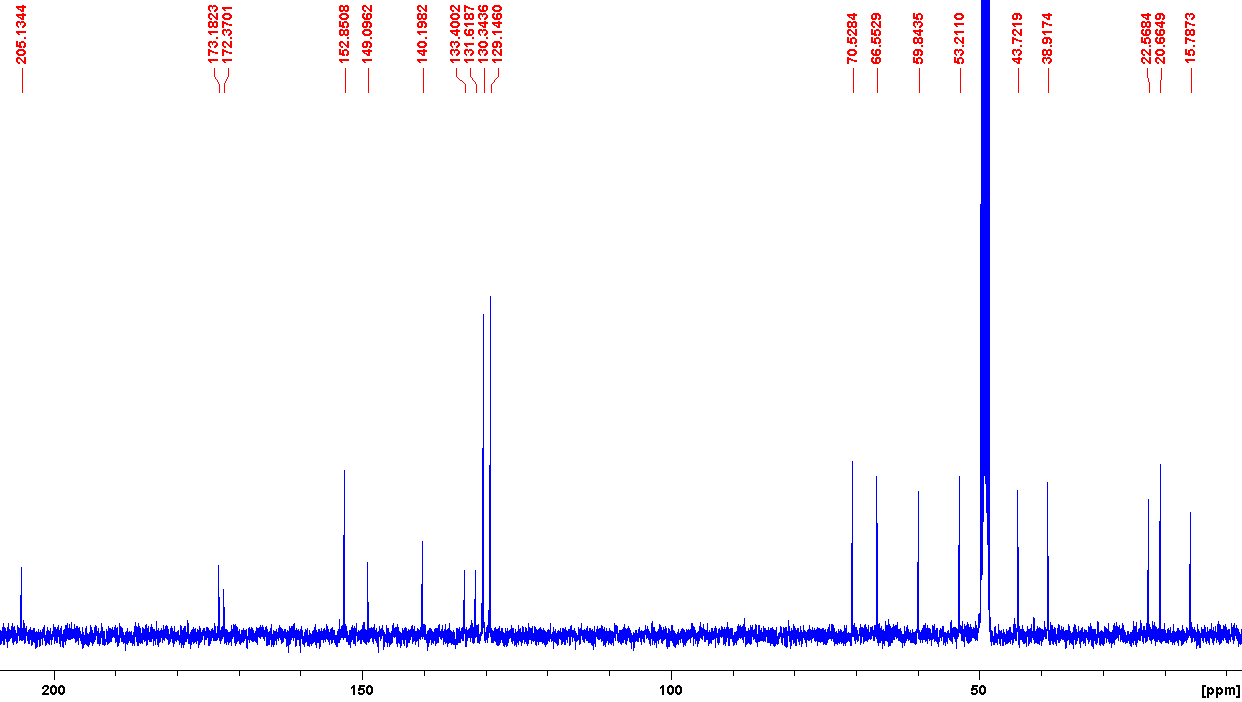


**Supplementary Figure 1. ^1^H and ^13^C NMR spectra of final compound 2 (NMR spectrum obtained in CD_3_OD)**

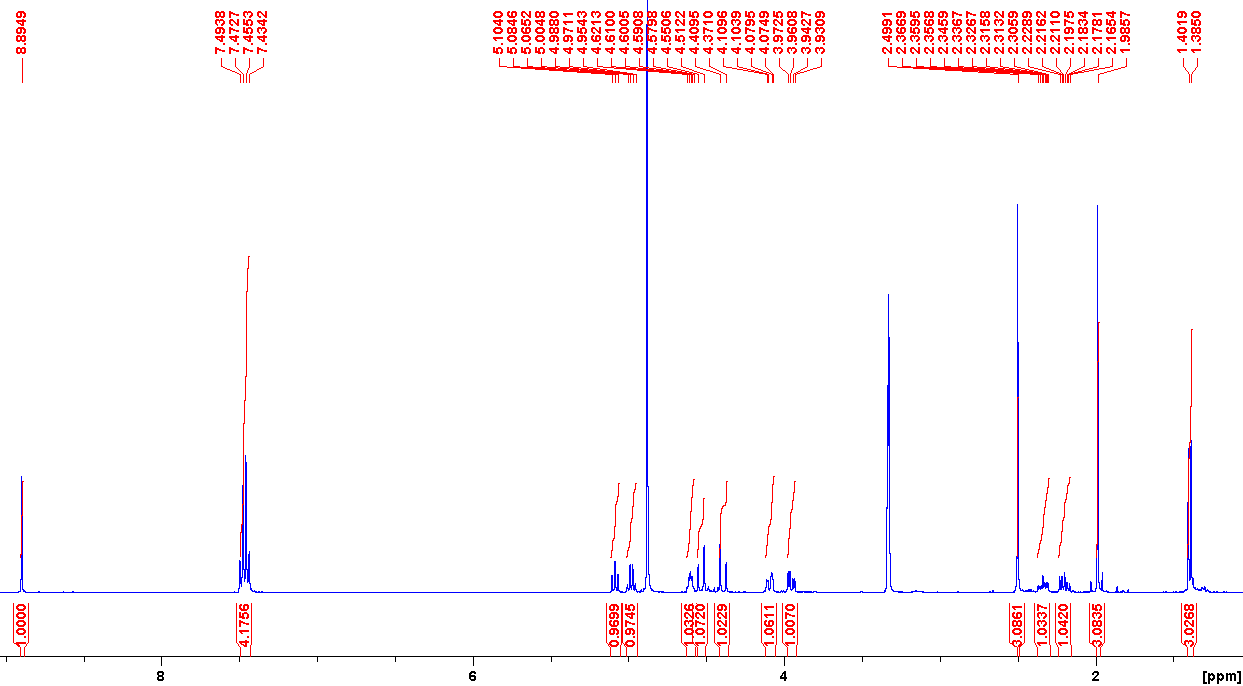


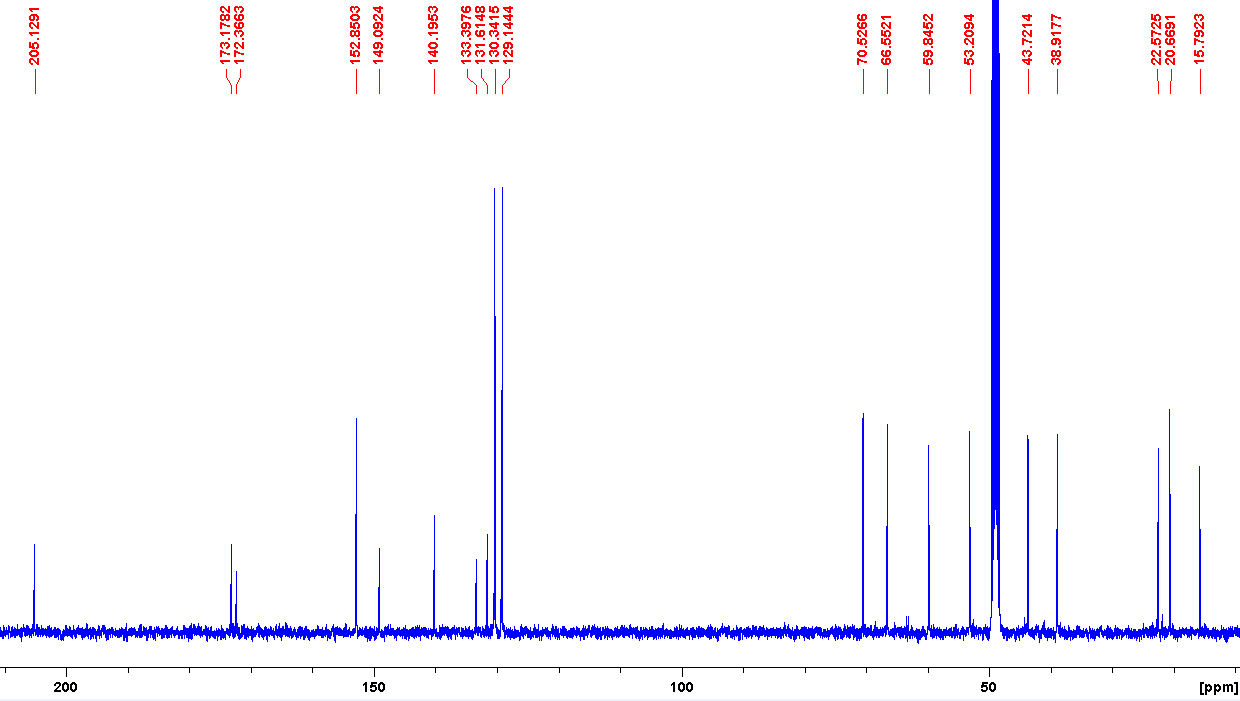


**Supplementary Figure 2. ^1^H and ^13^C NMR spectra of final compound 3 (NMR spectrum obtained in CD_3_OD)**

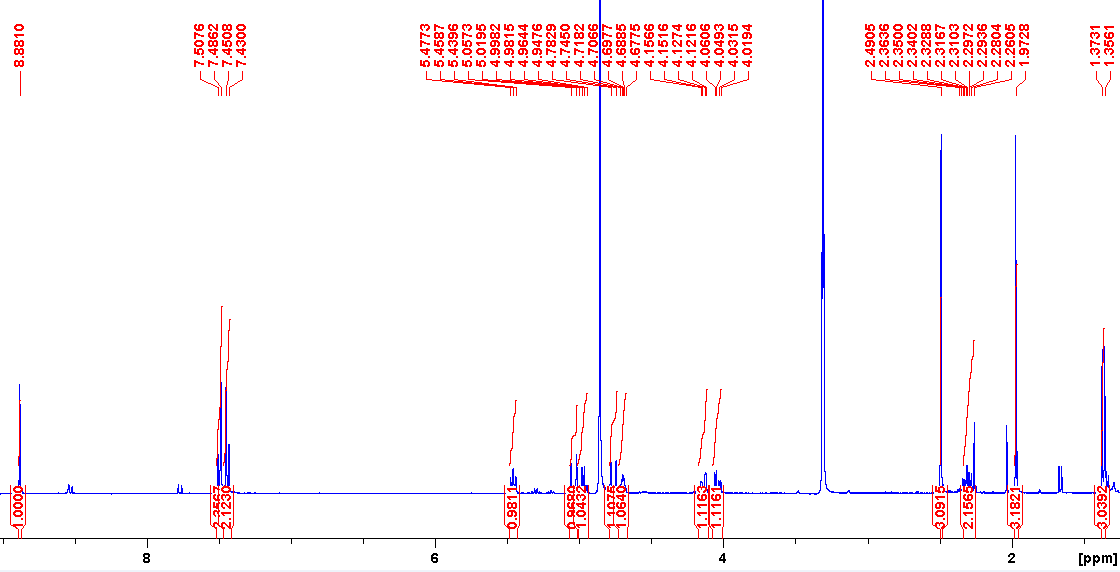


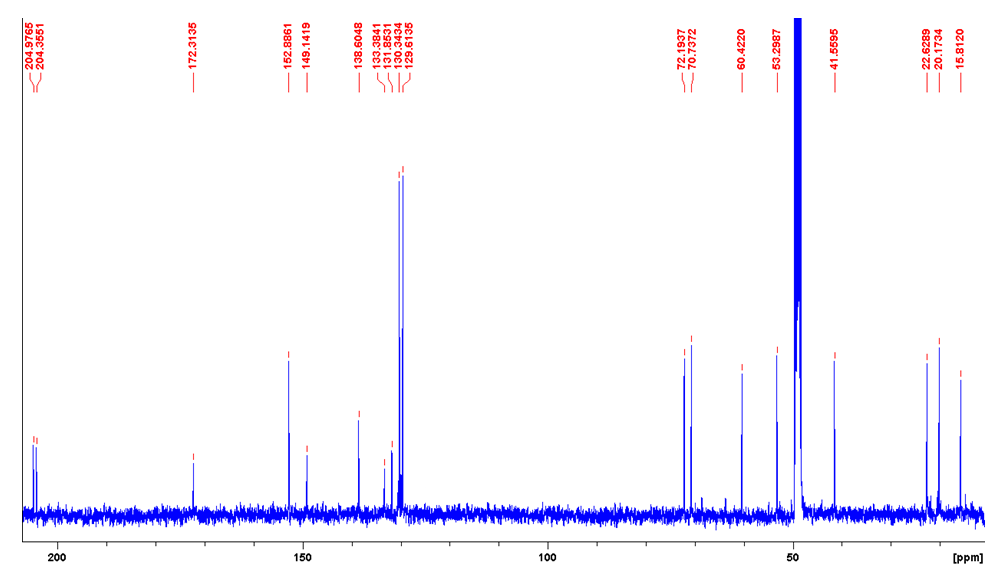


**Supplementary Figure 3. ^1^H and ^13^C NMR spectra of final compound 4 (NMR spectrum obtained in CD_3_OD)**

1. **FP competition assay protocol and data**

FP competitive binding experiments were performed on a PHERAstar FS (BMG LABTECH) in 384-well plates (Corning 3575), with an excitation wavelength (λ) at 485 nm and emission (λ)at 520 nm. Each well solution (15 µL) contained 15 nM of VBC protein, 10 nM of FAM-labelled HIF-1α peptide (FAM-DEALAHypYIPMDDDFQLRSF, *K*_d_ = 3 nM as measured by a direct FP titration) and decreasing concentrations of compound (14-point serial two-fold dilutions starting from 50 µM), in 100 mM Bis-tris, 100 mM NaCl, 1 mM DTT, pH 7. Control wells contained VBC and peptide in the absence of compound (maximum signal), and peptide in the absence of protein (background signal). Data were obtained in triplicate and the percentage of displacement was determined and graphed against log[VHL inhibitors]. Average IC_50_ values and the standard error of the mean (SEM) were determined for each titration using Prism 6. Dissociation constants *K*_d_ were back-calculated from the measured IC_50_ values using a displacement binding model, as described previously.^6^

1. **ITC protocols and data**

ITC experiments were carried in an ITC200 micro-calorimeter (GE Healthcare). Compounds **1-3** were diluted from DMSO stock solution to 600 µM and compound **4** to 1 mM in a buffer of 20 mM Bis-Tris propane, 150 mM NaCl, 1 mM DTT, pH 7. Compounds **1**-**4** were titrated against 60 or 100 µM VBC complex respectively, equilibrated in the same buffer. The final concentration of DMSO in each experiment was 3% (v/v). The titrations consisted of 20 injections of 2 µL of ligand solution at a rate of 0.5 µL/s at 120 s time intervals. An initial injection of ligand (0.4 µL) was made and discarded during data analysis. All experiments were performed at 25**°**C whilst stirring at 750 rpm. The data were fitted to a single-binding-site model using the Microcal LLC ITC200 Origin software provided by the manufacturer to obtain the stoichiometry *n*, the dissociation constant *K*_d_ and the enthalpy of binding Δ*H*.

1. **Quantification of ligands *trans* and *cis* isomers in solution.**

Each compound (7 mg) was solubilized in CD_3_OD and equilibrated overnight. The ^1^H NMR spectra was recorded on a Bruker DPX-400 Cryo spectrometer and analyzed using Bruker TopSpin 3.2 software package.^7^ The compounds acetamide methyl signals for the *cis* and *trans* isomers were fitted, and the value of the relative percentage of each isomer was determined from the relative areas of the calculated fits.

1. **X-ray crystallography protocol, data processing and refinement statistics**

**X-ray crystallography.**

The VBC ternary complex was purified and crystallized as described previously.^6,8^ Equal volume solutions of VBC (~5 mg/mL) and liquor solution were mixed in the hanging-drop vapor diffusion method at 18°C. The liquor solution contained 0.1 mM sodium cacodylate, pH 6.2-6.6, 16-18% polyethylene glycol 3350, 0.2 M magnesium acetate and 10 mM DTT. The drop was streaked with seeds of disrupted VBC crystals and a 2–3 mm layer of Al's Oil (Hampton Research) was applied on top of the liquor solution to slow the vapour diffusion rate. To obtain the structures of VHL inhibitors bound to VBC, crystals were soaked for 6h in a 3 mM solution of inhibitor in 3% DMSO, 12% isopropanol and 85% liquor solution. Crystals were screened using an in-house Rigaku M007HF x-ray generator and Saturn 944HG+ CCD detector. X-ray data were collected at 100 K at Diamond Light Source beamline I04-1. Indexing and integration of reflections was performed using XDS with the XDSGUI interface,^9^ and scaling and merging with AIMLESS in CCP4i.^10,11^ The isomorphous datasets were refined using REFMAC5^12,13^ and COOT^14^ using a template structure derived from the Protein Data Bank (PDB) entry 1vcb ref.^15^. Ligand structures and restraints were generated using the PRODRG server.^16^ The MOLPROBITY server was used to validate the geometry and steric clashes in the structures.^17^The structures have been deposited in the PDB with accession codes 6FMI, 6FMJ and 6FMK respectively and data collection and refinement statistics are presented in Supplementary Table 1.

**Supplementary Table 1.**

Crystallographic data processing and refinement statistics.

Values in parentheses are for the highest resolution shell.

| **Dataset** | **Compound 2** | **Compound 3** | **Compound 4** |
| --- | --- | --- | --- |
|  |  |  |  |
| **Synchrotron** | Diamond | Diamond | Diamond |
| Beamline | I04-1 | I04-1 | I04-1 |
| Wavelength (Å) | 0.9174 | 0.9174 | 0.9282 |
|  |  |  |  |
| **Processing statistics** |  |  |  |
| Space group | C222_1_ | P4_1_22 | P4_1_22 |
| Unit cell parameters |  |  |  |
| *a*,*b* (Å) | 64, 69.8 | 93.5 | 95 |
| *c* (Å) | 365.8 | 364 | 358 |
| Resolution limits (Å) | 47.18 - 2.80 (2.95- 2.80) | 48.97 - 2.51 (2.51 - 2.45) | 47.38 - 2.85 (2.85 - 2.75) |
| Total reflections | 101972 (14615) | 445402 (33487) | 250958 (26812) |
| Unique reflections | 20290 (2921) | 60839 (4397) | 44616 (4594) |
| Completeness (%) | 99.9 (99.9) | 100.0 (100) | 100.0 (100) |
| Multiplicity | 5.0 (5.0) | 7.3 (7.6) | 5.6 (5.8) |
| *R*_merge_ (%) | 12.3 (73.4) | 11.4 (85.5) | 9.3 (70.2) |
| *I*/σ(*I*) | 10.0 (2.4) | 10.6 (2.1) | 10.6 (2.1) |
| CC_1/2_ (%) | 99.6 (79.7) | 99.8 (84.8) | 99.8 (80.2) |
| Wilson *B* factor (Å^2^) | 38.0 | 38.2 | 43.8 |
| Mosaicity (°) | 0.14 | 0.08 | 0.09 |
|  |  |  |  |
| **Refinement statistics** |  |  |  |
| Resolution limits (Å) | 47.18-2.80  (2.87-2.80) | 48.97-2.45 (2.51-2.45) | 47.38-2.75  (2.82-2.75) |
| *R*_work_ (%) | 19.8 (28.7) | 21.7 (30.3) | 19.6 (28.4) |
| *R*_free_ (%) | 25.6 (31.8) | 26.6 (35.4) | 25.9 (37.0) |
| No. reflections | 19194 (1040) | 57673 (3059) | 42255 (2282) |
| No. test reflections | 1345 (80) | 4180 (201) | 3060 (172) |
| Model atoms | 5502 | 11679 | 11290 |
| Protein *B* factor (Å^2^) | 65.3 | 21.1 | 68.8 |
| Ligand *B* factor (Å^2^) | 50.2 | 38.4 | 54.7 |
| r.m.s.d. bonds (Å) | 0.007 | 0.007 | 0.008 |
| r.m.s.d. angles (°) | 1.17 | 1.17 | 1.19 |
|  |  |  |  |
| **Ramachandran plot** |  |  |  |
| Favoured (%) | 94.9 | 96.5 | 96.1 |
| Allowed (%) | 4.2 | 2.8 | 3.1 |
| Disallowed (%) | 0.9 | 0.7 | 0.8 |


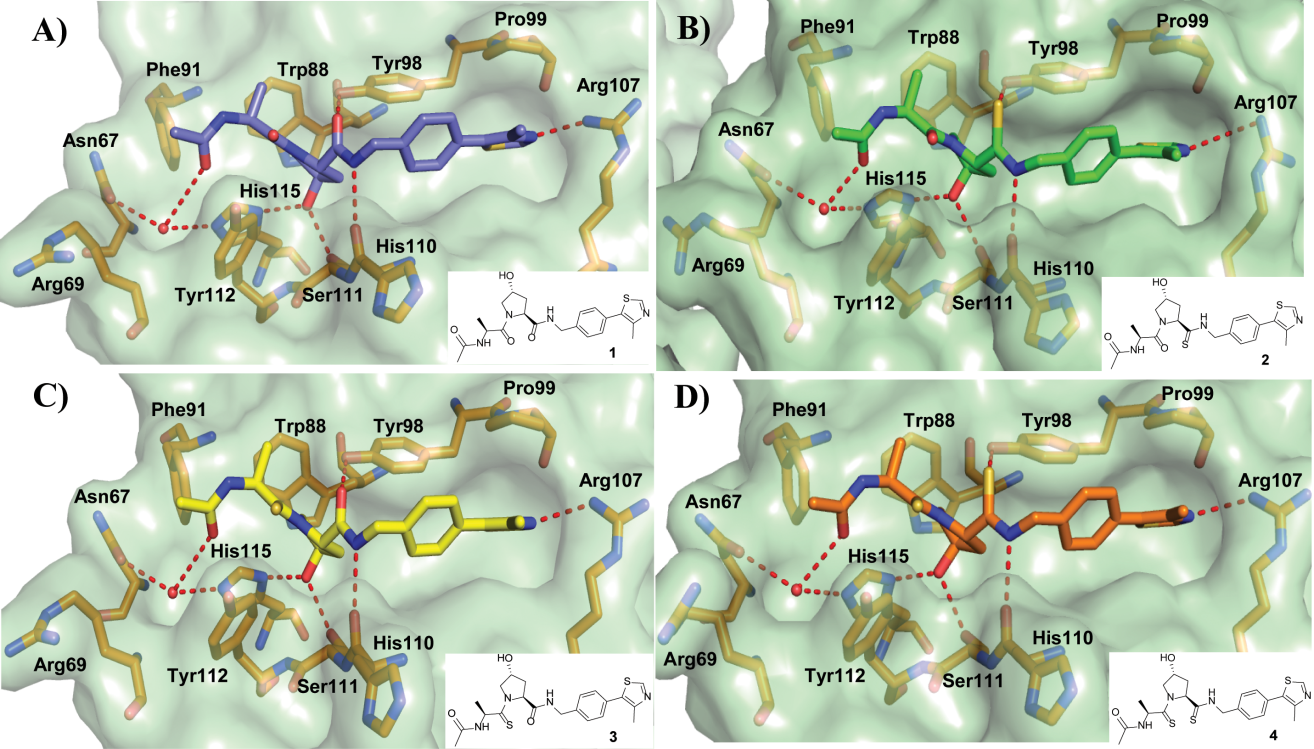


**Supplementary Figure 4.** Crystal structures of VBC in complex with A) compound **1**, purple carbons, 5NVY; B) **2**, green carbons, 6FMI; C) **3**, yellow carbons, 6FMJ; and D) **4**, orange carbons, 6FMK. VHL is shown as a pale green surface and the VHL residues forming the binding pocket as orange stick representations. Waters forming hydrogen bonds with the compounds are shown as red spheres. Hydrogen bond interactions between inhibitors, bound waters and VHL pocket residues are shown as dashed red lines.


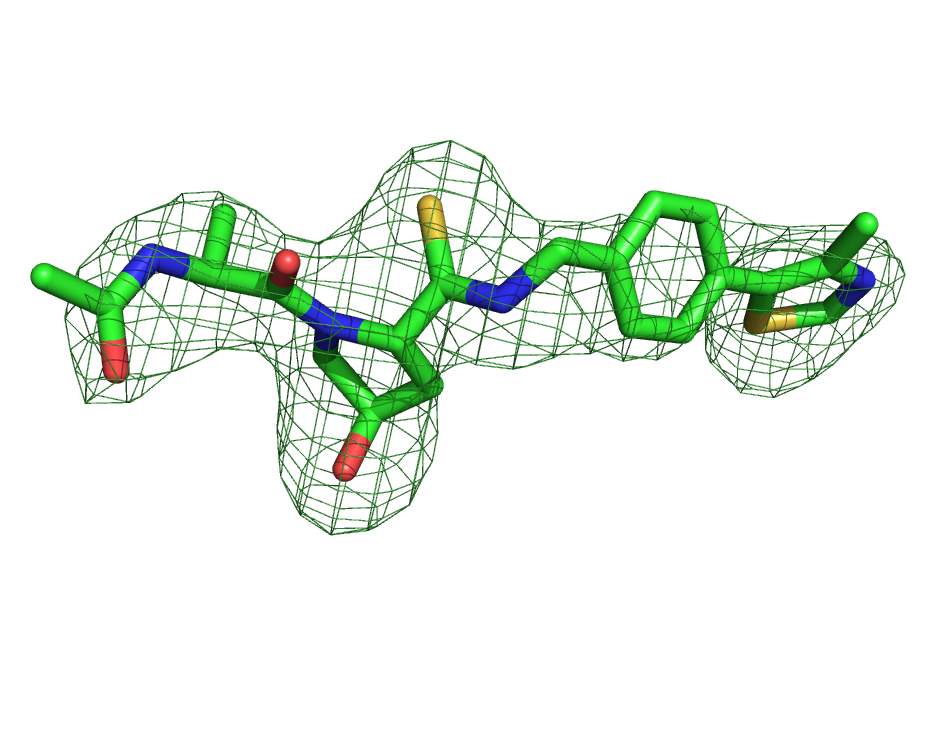


**A)**


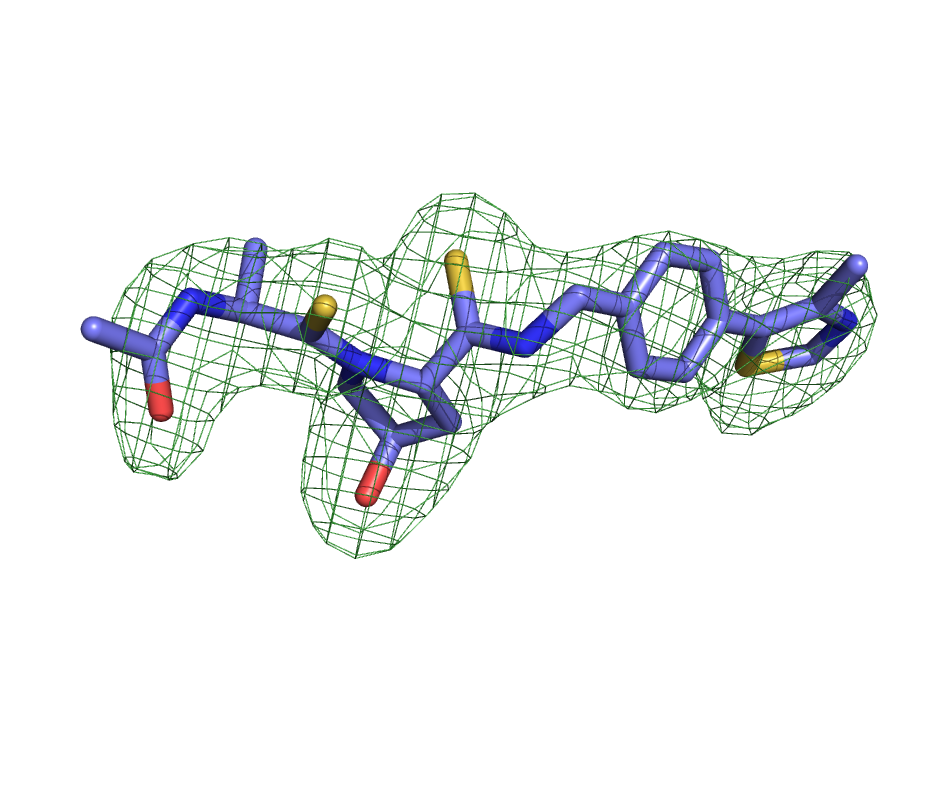

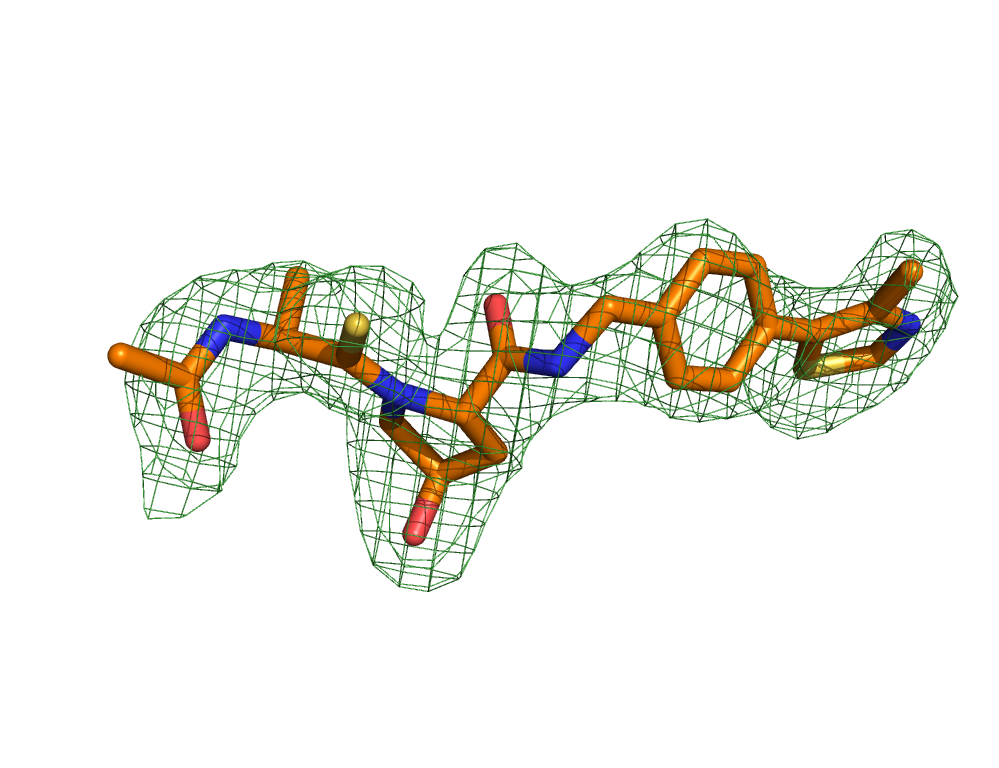


**C)**

**B)**

**Supplementary Figure 5.** Electron density at ligand binding sites. Compounds A) **2**; B) **3**; and C) **4** shown as sticks bound to VBC (not shown). An omit map (*F*_o_–*F*_c_) is shown in green and contoured at 3σ around each modelled ligand with a carve radius of 2.0 Å.

1. **Computational methods**

**Quantum mechanics calculations.** Model compounds **10–13** (see Supplementary Figure 6) were subjected to water phase all-atom energy minimization using local density functional theory (DFT) at the PBF (water) MN15-L/aug-cc-pVTZ(-F) level of theory in Jaguar 9.4 (Schrödinger Inc., LLC) and a tight self-consistent field (SCF) convergence criterion. The geometry optimization and frequency calculations were performed on the *endo* and *exo* puckers of pyrrolidine-containing compounds **10–13**. Frequency calculations of the optimized structures yielded no imaginary or low (> –40 cm^-1^) imaginary frequencies, indicating a true stationary point on the potential energy surface had been attained. The resulting SCF energies were corrected by the zero-point vibrational energy (ZPE) determined in the frequency calculations, and are listed in Supplementary Table 2. Optimized geometries were analyzed by natural bond order (NBO) theory using NBO 6.0^18^ at the same level of theory, as implemented in Jaguar.

**Molecular mechanics calculations.** The crystal structures of VBC in complex with compounds **1–4** (PDB codes 5NVY, 6FMI, 6FMJ and 6FMK, respectively) were used for the molecular modeling. Elongins B and C and all water molecules except for the one buried in VHL and directly involved in binding (Fig. 4B) were removed. Then, the complexes were prepared using the Protein Preparation Wizard (Schrödinger Inc.). Amino acid protonation states were assigned using PROPKA 3.0.^19,20^ The prepared systems were subjected to relative interaction energy prediction using the molecular mechanics with generalized-Born surface area (MM-GBSA) protocol in Prime 3.0 (Schrödinger Inc.), considering water solvation and a protein shell of 5.0 Å surrounding the compounds as flexible with constraints.

**Supplementary Figure 6.** Chemical structure of model compounds **10–13**.

**Supplementary Table 2.** Summary of energetic and geometric results of DFT calculations on model compounds **10–13** at the PBF (water) MN15-L/aug-cc-PVTZ(-F) level of theory.

| Compound | Hydroxyproline pucker | *E* (hartree) | ZPE (kcal/mol) | *E*_corrected_ (hartree) | Δ*E*_exo-endo_ (kcal/mol) | Hydroxyproline pucker (%) | *E_n_*_→_*_π_*_*_ (kcal/mol) | Weighted *E_n_*_→_*_π_*_*_ (kcal/mol) | Pyramidalization of the acceptor C (Å) | Imaginary frequencies |
| --- | --- | --- | --- | --- | --- | --- | --- | --- | --- | --- |
| 10 | endo | -667.9741 | 134.51 | -667.7597 | -0.37 | 35 | 0.3 | 2.1 | 0.015 | 0 |
|  | exo | -667.9746 | 134.48 | -667.7603 |  | 65 | 3.1 |  | 0.013 | 0 |
| 11 | endo | -990.9218 | 133.00 | -990.7099 | -0.29 | 38 | 0.3 | 2.0 | 0.018 | 1*^a^* |
|  | exo | -990.9223 | 132.98 | -990.7104 |  | 62 | 3.1 |  | 0.010 | 1*^b^* |
| 12 | endo | -990.9320 | 133.20 | -990.7198 | -0.42 | 33 | 1.1 | 2.6 | 0.024 | 0 |
|  | exo | -990.9328 | 133.26 | -990.7205 |  | 67 | 3.4 |  | 0.026 | 0 |
| 13 | endo | -1,313.8793 | 131.71 | -1,313.6694 | -0.48 | 31 | 1.5 | 3.4 | 0.023 | 1*^c^* |
|  | exo | -1,313.8800 | 131.69 | -1,313.6701 |  | 69 | 4.3 |  | 0.028 | 0 |

*^a^* Frequency = –9.5 cm^-1^

*^b^* Frequency = –16.6 cm^-1^

*^a^* Frequency = –30.4 cm^-1^

**Supplementary Figure 7.** Structure of compounds A) **10**, B) **11**, C) **12**, and D) **13** at the minimized energy state, in (top) C*^4^* *endo* and (bottom) C*^4^* *exo* conformation. DFT calculations were carried out at the PBF (water) MN15-L/aug-cc-pVTZ(-F) level of theory.


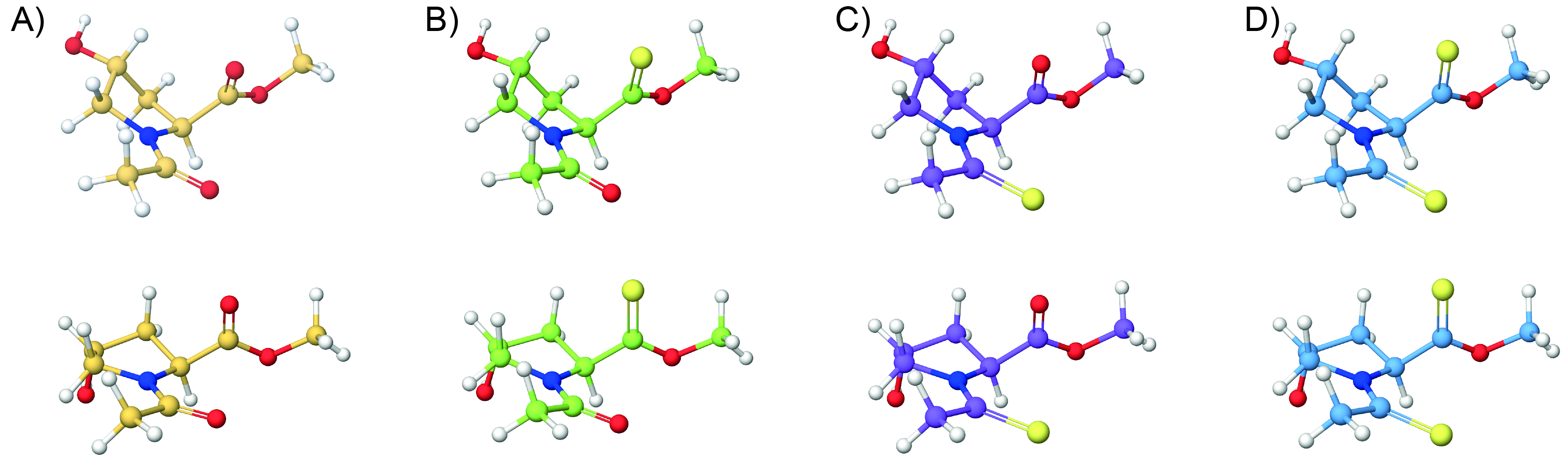


**Supplementary Figure 8.** Predicted interaction energy (*E*_MM-GBSA_) versus experimental Gibbs free energy (Δ*G*) for compounds **1–4** binding to VBC.


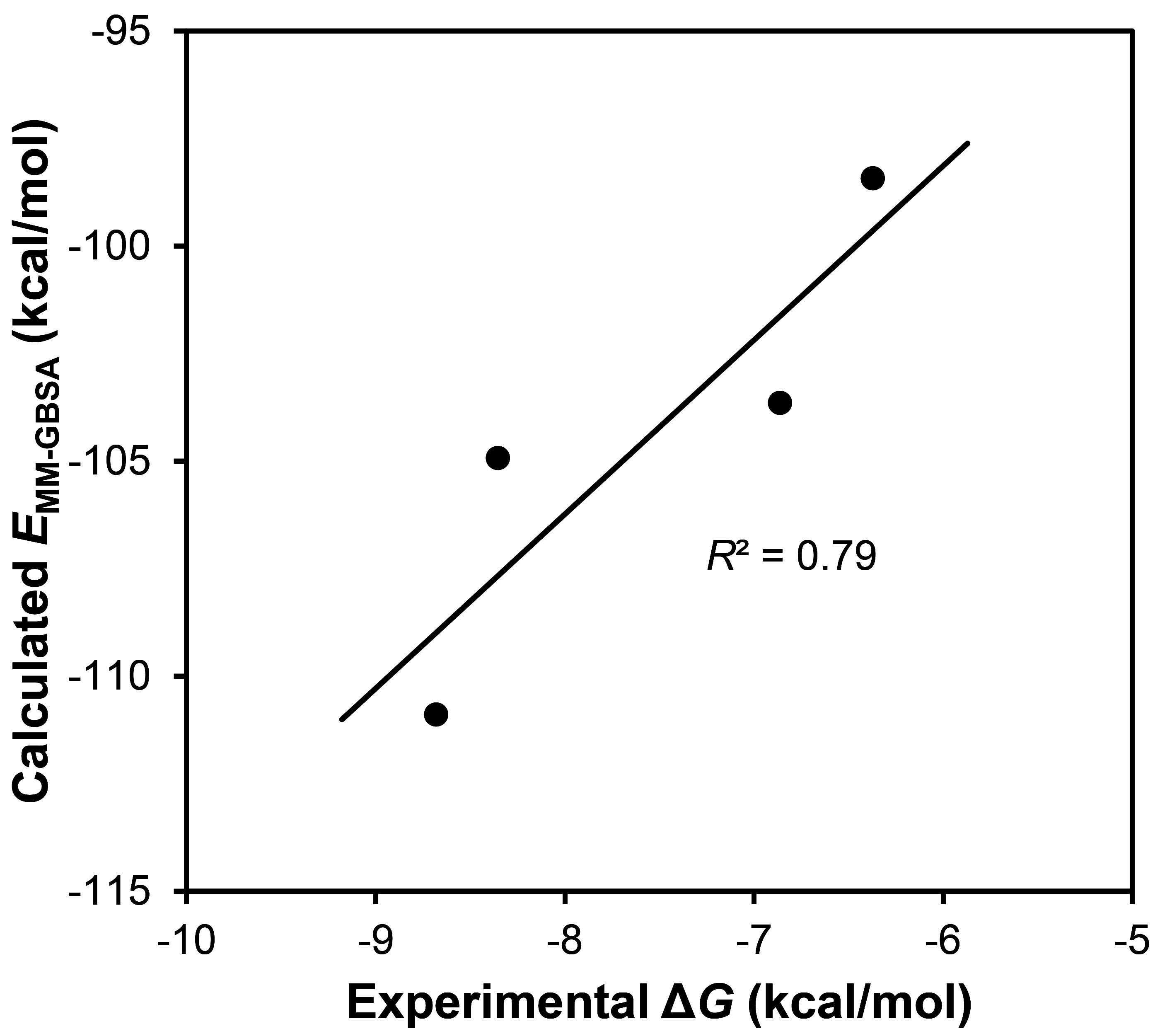


**References**

1. Frost, J.; Galdeano, C.; Soares, P.; Gadd, M. S.; Grzes, K. M.; Ellis, L.; Epemolu, O.; Shimamura, S.; Bantscheff, M.; Grandi, P.; Read, K. D.; Cantrell, D. A.; Rocha, S.; Ciulli, A. *Nat. Commun.* **2016**, *7*, 13312.

2. Galdeano, C.; Gadd, M. S.; Soares, P.; Scaffidi, S.; Van Molle, I.; Birced, I.; Hewitt, S.; Dias, D. M.; Ciulli, A. *J. Med. Chem.* **2014**, *57*, 8657–8663.

3. Soares, P.; Gadd, M. S.; Frost, J.; Galdeano, C.; Ellis, L.; Epemolu, O.; Rocha, S.; Read, K. D.; Ciulli, A. *J. Med. Chem.* **2018**, *61*, 599-618.

4. Mukherjee, S.; Verma, H.; Chatterjee, *J*. *Org. Lett.* **2015**, *17*, 3150–3153.

5. Ashraf Shalaby, M.; Grote, C. W.; Rapoport, H. *J. Org. Chem.* **1996**, *61*, 9045–9048.

6. Van Molle, I.; Thomann, A.; Buckley, D. L.; So, E. C.; Lang, S.; Crews, C. M.; Ciulli, A. *Chem. Biol.* **2012**, *19*, 1300–1312.

7. Bruker Topspin Version 3.2, Brruker, Banner Lane, Coventry, CV4 9GH .

8. Buckley, D. L.; Van Molle, I.; Gareiss, P. C.; Tae, H. S.; Michel, J.; Noblin, D. J.; Jorgensen, W. L.; Ciulli, A.; Crews, C. M. *J. Am. Chem. Soc.*  **2012**, *134*, 4465–4468.

9. Kabsch, W. *Acta Crystallogr. Sect. D Biol. Crystallogr.* **2010**, *66*, 125–132.

10. Evans, P. R.; Murshudov, G. N. *Acta Crystallogr. Sect. D Biol. Crystallogr.* **2013**, *69*, 1204–1214.

11. Winn, M. D.; Ballard, C. C.; Cowtan, K. D.; Dodson, E. J.; Emsley, P.; Evans, P. R.; Keegan, R. M.; Krissinel, E. B.; Leslie, A. G. W.; McCoy, A.; McNicholas, S. J.; Murshudov, G. N.; Pannu, N. S.; Potterton, E. A.; Powell, H. R.; Read, R. J.; Vagin, A.; Wilson, K. S. Overview of the CCP4 suite and current developments. *Acta Crystallogr. Sect. D Biol. Crystallogr.* **2011**, *67*, 235–242.

12. Murshudov, G. N.; Skubák, P.; Lebedev, A. A.; Pannu, N. S.; Steiner, R. A.; Nicholls, R. A.; Winn, M. D.; Long, F.; Vagin, A. A. *Acta Crystallogr. Sect. D Biol. Crystallogr.* **2011**, *67*, 355–367.

13. Murshudov, G. N.; Vagin, A. A.; Dodson, E. J. Refinement of macromolecular structures by the maximum-likelihood method. *Acta Crystallogr. Sect. D Biol. Crystallogr.* **1997**, *53*, 240–255.

14. Emsley, P.; Lohkamp, B.; Scott, W. G.; Cowtan, K. *Acta Crystallogr. Sect. D Biol. Crystallogr.* **2010**, *66*, 486–501.

15. Stebbins, C. E.; Kaelin, W. G.; Pavletich, N. P. *Science* **1999**, *284*, 455–461.

16. Schüttelkopf, A. W.; Van Aalten, D. M. F. *Acta Crystallogr. Sect. D Biol. Crystallogr.* **2004**, *60*, 1355–1363.

17. Chen, V. B.; Arendall, W. B.; Headd, J. J.; Keedy, D. A.; Immormino, R. M.; Kapral, G. J.; Murray, L. W.; Richardson, J. S.; Richardson, D. C. *Acta Crystallogr. Sect. D Biol. Crystallogr.* **2010**, *66*, 12–21.

18. E. D. Glendening, J. K. Badenhoop, A. E. Reed, J. E. Carpenter, J. A. Bohmann, C. M. Morales, C. R. Landis, and F. W. No. *(Theoretical Chem. Institute, Univ. Wisconsin, Madison, WI, 2013.*

19. Olsson, M. H. M.; Søndergaard, C. R.; Rostkowski, M.; Jensen, J. H. *J. Chem. Theory Comput.* **2011**, *7*, 525–537.

20. Søndergaard, C. R.; Olsson, M. H. M.; Rostkowski, M.; Jensen, J. H. *J. Chem. Theory Comput.* **2011**, *7*, 2284–2295.
